# Synthesis and DPPH Radical Scavenging Activity of Prenylated Phenol Derivatives

**DOI:** 10.3390/molecules17010556

**Published:** 2012-01-06

**Authors:** Mauricio Osorio, Jacqueline Aravena, Alejandra Vergara, Lautaro Taborga, Evelyn Baeza, Karen Catalán, Cesar González, Marcela Carvajal, Héctor Carrasco, Luis Espinoza

**Affiliations:** 1 Departamento de Química, Universidad Técnica Federico Santa María, Av. España N° 1680, Valparaíso, 2390123, Chile; 2 Departamento de Ciencias Químicas, Universidad Andrés Bello, Campus Viña del Mar, Los Fresnos N 52, Viña del Mar, 2561156, Chile

**Keywords:** prenylated phenols, electrophilic aromatic substitution, radical scavenging activity

## Abstract

The synthesis of twenty six prenylated phenols derivatives is reported. These compounds were obtained under mild conditions via Electrophilic Aromatic Substitution (*EAS*) coupling reactions between phenol derivatives containing electron-donor subtituents and 3-methyl-2-buten-1-ol using BF_3_·OEt_2_. Dialkylations were also produced with this method. The formation of a chroman ring by intramolecular cyclization between a sp^2^ carbon from the prenyl group with the hydroxyl substituent in the *ortho* position occurred with some phenols. All the synthesized compounds were evaluated as antioxidants according to a DPPH radical scavenging activity assay. IC_50_ values of five synthesized compounds indicated they were as good antioxidants as Trolox™.

## 1. Introduction

Prenylated phenols constitute an interesting group of marine natural products, for which a wide variety of biological activities have been described, including anti-inflammatory [1,2], antifungal [3], anti-HIV [4], anti-Alzheimer activity [5] and most frequently, antineoplastic properties [6,7]. *ortho*-Prenylated phenols play an important role in mediating many biological processes. For instance, prenylated ubiquinones are essential in cellular respiration [8]. Clearly, such an important structural motif needs a general strategy for its preparation, particularly for systems in which other aromatic hydroxyl residues are differentiated, as is often the case with therapeutic natural products containing this pharmacophore.

As a part of our ongoing interest in developing new and efficient antitumour agents, we recently reported the synthesis of two new hemisynthetic diterpenylhydroquinones from natural *ent*-labdanes by coupling between an arene nucleus and an allylic alcohol, which showed important activity in the inhibition of the growth of cancerigenous cells [9,10]. This kind of molecules are structural analogs of terpenylquinones and terpenylhydroquinones which are characteristic marine metabolites frequently isolated from alga and/or sponge [11,12].

Terpenylphenols are isolated from natural sources in very low yield, and for that reason during the last few decades, considerable research effort has been focused on obtaining these compounds by synthesis. The most recurrent strategies used for synthesizing these compounds involve, as a first step, the separate preparation of the appropriate terpenyl fragments and aromatic nucleus. The crucial step is the attachment of the aromatic synthon to the terpenyl skeleton [13]. There are many publications that report different methods for accomplishing these coupling reactions in the synthesis of prenylphenols, but the most interesting method for us is the Electrophilic Aromatic Substitution (*EAS*) reaction between a phenol and the corresponding prenol by using BF_3_·OEt_2_ because of its simplicity and mild reaction conditions [8,14].

Endogenous free radicals generated during body metabolism play an important role in the human health by causing several diseases including cancer, hypertension, heart attack and diabetes. Oxidation of low-density lipoprotein (LDL) is thought to play a central role in atherosclerosis. Reactive oxygen and nitrogen species as singlet oxygen, superoxide radicals, peroxyl radicals, hydroxyl radicals, and peroxynitrite can react with critical cellular components such as DNA, lipids, and proteins leading to tissue injury and contributing to chronic diseases. In recent years much attention has been focused on the use of natural dietary antioxidants as an effective protection against diseases related to oxidative processes [15,16,17]. A series of prenylated phenols derivatives were synthesized and their antioxidant activities (DPPH radical scavenging activity) were evaluated. 

## 2. Results and Discussion

### 2.1. Synthesis

**Scheme 1:**
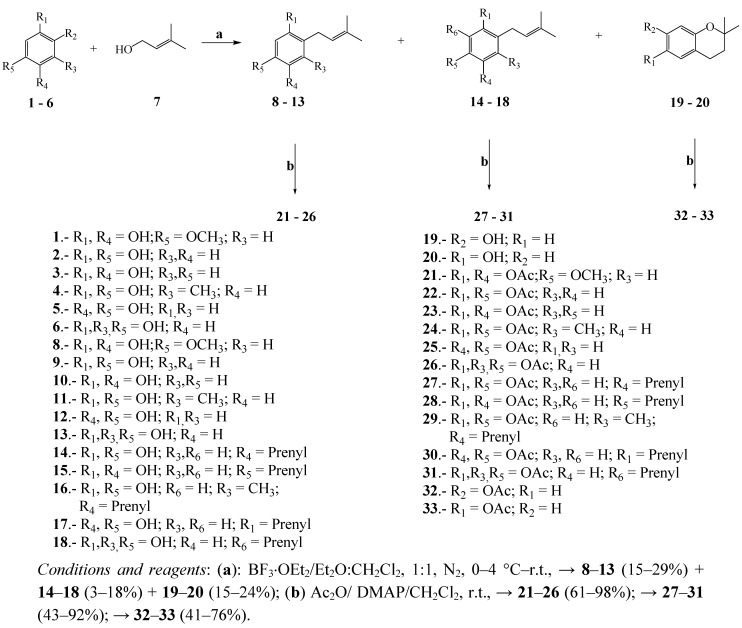
Synthesis of prenylated phenols and their acetylated derivatives in two steps.

The synthesis of prenylated phenols was carried out in one step via the *EAS* mechanism as shown in Scheme 1. The reaction of different phenols **1**–**6** with prenol in the presence of BF_3_ etherate in a 1:1 mixture of diethyl ether/CH_2_Cl_2_ produced prenylated phenols, dialkylated phenols and chromans in moderate yields (15–29%, 3–11%, 15–24% respectively). The higher yields were found in acetylation reactions from prenylated phenols (41–98%).

The structures of the monoalkylated and dialkylated phenols were mainly established by ^1^H, ^13^C, DEPT-135, gs-2D HSQC and gs-2D HMBC NMR techniques. The presence of aromatic signals in the ^1^H-NMR data spectra and their integration showed the positions of the alkylations with prenyl groups, *i.e.*, the monoalkylated compound **9** showed a doublet at δ 6.92 (1H, *J* = 7.8 Hz, Ar*H*-5), a doublet at δ 6.37 (1H, *J* = 8.1 Hz, Ar*H*-6) and a singlet at δ 6.36 (1H, Ar*H*-2) but the dialkylated compound **14** showed a singlet at δ 6.78 (1H, Ar*H*-5) and a singlet at δ 6.33 (1H, Ar*H*-2), confirming the substitution of the aromatic protons at H-4 and H-6 positions by prenyl groups.

It was found in results not shown that chromans were obtained mainly when equimolar amounts of catalyst were used with resorcinol (**2**) and hydroquinone (**3**) as starting material. Similar behavior was reported with other phenols [18]. The structural determination of the compounds **19** and **20** was mainly accomplished by ^1^H- and ^13^C-NMR data spectra. The ^1^H-NMR spectrum showed the existence of aliphatic protons at δ = 1.34 (**19**) and 1.32 (**20**), indicating the presence of two magnetically equivalent CH_3_ groups. Also two triplets at δ = 2.70 and 1.79 for **19**; and δ = 2.69 and 1.76 for **20**, indicating the existence of CH_2_ groups typical from chromans (C-3 and C-4). By-products were not obtained in acetylation reactions.

### 2.2. Antioxidant Activity

The results obtained from antioxidant activities assays are shown in Table 1. All compounds were compared with Trolox™. All acetylated compounds were inactive. The prenylated phenols **8**, **10**, **12**, **15** and **17** have antioxidant similar to Trolox™. Dialkylated compounds largely display better activities than monoalkylated compounds for the same phenol.

**Table 1:** Screening results of DPPH radical scavenging activity of phenols **1–6** and prenylated phenols derivatives **8**–**33**.

Compound	IC_50_μM ± SD	Compound	IC_50_μM ± SD
1	18.56 ± 1.55	18	45.36 ± 0.86
2	NA	19	NA
3	28.75 ± 2.46	20	35.63 ± 2.87
4	173.44 ± 5.12	21	NA
5	22.07 ± 0.02	22	NA
6	NA	23	NA
8	23.68 ± 0.34	24	NA
9	81.22 ± 4.75	25	NA
10	18.68 ± 1.48	26	NA
11	60.34 ± 3.04	27	NA
12	21.38 ± 1.80	28	NA
13	114.54 ± 3.02	29	NA
14	50.94 ± 4.29	30	NA
15	21.19 ± 1.39	31	NA
16	48.32 ± 1.14	32	NA
17	19.06 ± 1.69	33	NA
Trolox™	22.04 ± 2.11		

Antioxidant activity are shown as IC_50_ values in µM concentrations; NA = no activity. All compounds were analyzed in triplicate and the results expressed as average ± standard deviation.

## 3. Experimental

### 3.1. General

All chemical reagents purchased (Merck or Aldrich) were of the highest commercially available purity and were used without previous purification. Melting points (mp: °C) were measured on a melting point apparatus Stuart-Scientific SMP3 and are uncorrected. IR spectra were recorded as a methylene chloride solution or KBr disk (compound **13**) in a Thermo Scientific Nicolet 6700 FT-IR spectrometer and frequencies are reported in cm^−1^. Low resolution mass spectra were recorded on a Thermo Scientific, Trace GC Ultra, ISQ mass spectrometer at 70 eV ionizing voltage and are given as *m/z* (% rel. int.) and these were measured in acetylated derivatives only. ^1^H-, ^13^C-(DEPT 135 and DEPT 90), HSQC and 2D HMBC spectra were recorded in CDCl_3_ solutions and referenced to the residual peaks of CHCl_3_ at δ 7.26 ppm and δ 77.0 ppm for ^1^H and ^13^C, respectively, on a Bruker Avance 400 Digital NMR spectrometer, operating at 400.1 MHz for ^1^H and 100.6 MHz for ^13^C. Chemical shifts are reported in δ ppm and coupling constants (*J*) are given in Hz. Silica gel (Merck 200–300 mesh) was used for C.C. and silica gel plates HF-254 for TLC. TLC spots were detected by both under UV lamp and heating after spraying with 25% H_2_SO_4_ in H_2_O. Antioxidant determinations were performed in a Thermo Scientific Multiskan FC 96-well plate photometer.

### 3.2. General Procedure for Preparation of Prenylated Phenols

A solution of phenol (4.5 mmol) and 3-methyl-2-buten-1-ol (**7**, 9.0 mmol) was placed in a round bottom flask and dissolved in dry 1:1 ethyl ether-dichloromethane (25 mL). Under nitrogen gas and with vigorous stirring, a solution of BF_3_ etherate (0.9 mmol) in dry 1:1 ethyl ether-dichloromethane (10 mL) was added dropwise to the solution cooled to 0–5 °C. Then the reaction mixture was allowed to warm up to room temperature and the stirring continued. After 48 h, milled ice was added to the reaction mixture and it was extracted with methylene chloride. Then, the organic layer was separated and a new extraction with ethyl acetate was done. The organic solutions obtained after extractions were mixed and dried over anhydrous sodium sulphate and filtered, the solvent was evaporated under reduced pressure. After, the mixture was subjected to silica gel flash column chromatography (ethyl acetate, petroleum ether) to obtain pure products.

#### 3.2.1. *1,4-Dihydroxy-2-methoxy-5-(3-methyl-2-buten-1-yl) Benzene* (**8**)

Compound **8** was obtained from 2-methoxy-1,4-dihydroxybenzene (**1**) as described above. The crude mixture was purified using petroleum ether-ethyl acetate (80:20) as the mobile phase to afford the title compound as an orange solid (126 mg, 24%); mp: 102–103 °C; IR (solution): ν_max_ 3210 (OH), 1621 (C=C aromatic), 1434, 1190; ^1^H-NMR (CDCl_3_): 6.67 (s, 1H, Ar*H*-6); 6.43 (s, 1H, Ar*H*-3); 5.28 (br. t, *J* = 7.2 Hz, 1H, CC*H*CH_2_); 5.13 (s, 1H, ArC-1-O*H*) 4.79 (s, 1H, ArC-4-O*H*); 3.43 (s, 3H, ArC-2-OC*H*_3_); 3.25 (d, *J* = 7.2 Hz, 2H, C=CHC*H*_2_); 1.77 [s, 6H, CHC(C*H*_3_)_2_]; ^13^C-NMR (CDCl_3_): 17.8 (*C*H_3_CH_3_C=CH_2_-); 25.7 (CH_3_*C*H_3_C=CH_2_-); 29.3 (CCH*C*H_2_-); 56.1 (*C*H_3_O-); 100.3 (Ar*C*H-3); 115.3 (Ar*C*H-6); 118.5 (Ar*C*-5); 121.9 (C=*C*HCH_2_); 134.8 (*C*=CHCH_2_); 139.3 (Ar*C*-1); 145.4 (Ar*C*-2); 147.5 (Ar*C*-4).

#### 3.2.2. *1,3-Dihydroxy-4-(3-methyl-2-buten-1-yl) Benzene* (**9**)

Compound **9** was obtained from resorcinol (**2**) as described above. The crude mixture was purified using petroleum ether-ethyl acetate (70:30) as the mobile phase to afford compound **9** as an orange semi-solid (186.4 mg, 23%); IR (solution): ν_max_ 3362 (OH), 2968 (alkane C-H), 2912 (C-H alkane), 1604 (C=C aromatic), 1518, 1451, 1157; ^1^H-NMR (CDCl_3_): 6.92 (d, 1H, *J* = 7.8 Hz, Ar*H*-5); 6.37 (d, 1H, *J* = 8.1 Hz, Ar*H*-6); 6.36 (s, 1H, Ar*H*-2); 5.29 (br. t, *J* = 6.7 Hz, 1H, CC*H*CH_2_); 3.26 (d, *J* = 7.0 Hz, 2H, C=CHC*H*_2_); 1.75 (s, 3H, CHCCH_3_C*H*_3_); 1.74 (s, 3H, CHCC*H*_3_CH_3_). ^13^C-NMR (CDCl_3_): 17.7 (*C*H_3_CH_3_C=CH_2_-); 25.7 (CH_3_*C*H_3_C=CH_2_-); 28.7 [(CH_3_)_2_CCH*C*H_2_-]; 103.3 (Ar*C*-2); 107.7 (Ar*C*-6); 119.6 (Ar*C*-4); 122.2 [(CH_3_)_2_C=*C*HCH_2_]; 130.5 (Ar*C*-5); 134.2 [(CH_3_)_2_*C*=CHCH_2_]; 154.6 (Ar*C*-1); 154.7 (Ar*C*-3).

#### 3.2.3. *1,4-Dihydroxy-2-(3-methyl-2-buten-1-yl) Benzene* (**10**)

Compound **10** was obtained from hydroquinone (**3**) as described above. The crude mixture was purified using petroleum ether-ethyl acetate (80:20) as the mobile phase to afford compound **10** as colorless needles (165.8 mg, 21%); mp: 102–104 °C, lit. [19], 100–101 °C; IR (solution): ν_max_ 3228 (OH), 1654 (C=C Aromatic), 1560, 1452, 1194; ^1^H-NMR (CDCl_3_): 6.68 (d, 1H, *J* = 8.5 Hz, Ar*H*-6); 6.61 (d, 1H, *J* = 3.0 Hz, Ar*H*-3); 6.58 (dd, *J* = 8.4 and 3.0 Hz, 1H, Ar*H*-5); 5.29 (br. t, *J* = 7.3 Hz, 1H, C=C*H*CH_2_); 3.29 (d, *J* = 7.2 Hz, 2H, C=CHC*H*_2_); 1.77 [s, 6H, CHC(C*H*_3_)_2_]; ^13^C-NMR (CDCl_3_): 17.8 (*C*H_3_CH_3_C=CH-); 25.8 (CH_3_*C*H_3_C=CH-); 29.7 [(CH_3_)_2_CCH*C*H_2_-]; 113.7 (Ar*C*-5); 116.4 (Ar*C*-6); 116.6 (Ar*C*-3); 121.4 ((CH_3_)_2_C=*C*HCH_2_); 128.2 (Ar*C*-2); 134.9 [(CH_3_)_2_*C*=CHCH_2_]; 148.1 (Ar*C*-1); 149.3 (Ar*C*-4).

#### 3.2.4. *1,3-Dihydroxy-5-methyl-4-(3-methyl-2-buten-1-yl) Benzene* (**11**)

Compound **11** was obtained from orcinol (**4**) as described above. The crude mixture was purified using petroleum ether-ethyl acetate (70:30) as the mobile phase to afford compound **11** as a reddish semi-solid (215.4 mg, 29%); IR (solution): ν_max_ 3218 (OH), 2964 (C-H alkane), 2923 (C-H alkane), 1610 (C=C aromatic), 1474, 1318, 1142; ^1^H-NMR (CDCl_3_): 6.26 (d, 1H, *J* = 2.0 Hz, Ar*H*-2); 6.21 (d, 1H, *J* = 2.1 Hz, Ar*H*-6); 5.14 (m, 2H, O*H*, C=C*H*CH_2_); 4.65 (s, 1H, O*H*) 3.28 (d, *J* = 6.8 Hz, 2H, C=CHC*H*_2_); 2.23 (s, 3H, Ar-C*H*_3_); 1.80 (s, 3H, C*H*_3_CH_3_C=CH_2_-); 1.73 (s, 3H, CH_3_C*H*_3_C=CH_2_-); ^13^C-NMR (CDCl_3_): 17.9 (*C*H_3_CH_3_C=CH-); 20.1 (CH_3_*C*H_3_C=CH-); 25.2 [(CH_3_)_2_CCH*C*H_2_-]; 25.7 (Ar-*C*H_3_-5); 101.0 (Ar*C*H-2); 109.7 (Ar*C*H-6) 117.9 (Ar*C*-4); 122.1 [(CH_3_)_2_C=*C*HCH_2_]; 133.9 [(CH_3_)_2_*C*=CHCH_2_]; 138.5 (Ar*C*-5); 154.2 (Ar*C*-1); 155.3 (Ar*C*-3).

#### 3.2.5. *1,2-Dihydroxy-4-(3-methyl-2-buten-1-yl) Benzene* (**12**)

Compound **12** was obtained from pyrocatechol (**5**) as described above. The crude mixture was purified using petroleum ether-ethyl acetate (70:30) as the mobile phase to afford compound **12** as a colorless solid (201.9 mg, 25%); mp: 58–61 °C; IR (solution): ν_max_ 3364 (OH), 2924 (C-H alkane), 2853 (C-H alkane), 1603 (C=C aromatic), 1518, 1452, 1376, 1280; ^1^H-NMR (CDCl_3_): 6.77 (d, 1H, *J* = 8.1 Hz, Ar*H*-6); 6.70 (d, 1H, *J* = 1.3 Hz, Ar*H*-3); 6.61 (dd, 1H, *J* = 7.4 Hz and 1.2 Hz, Ar*H*-5); 5.45 (s, 1H, O*H*) 5.37 (s, 1H, O*H*); 5.28 (br. t, *J* = 7.3 Hz, 1H, CC*H*CH_2_); 3.22 (d, *J* = 7.8 Hz, 2H, C=CHC*H*_2_); 1.74 (s, 3H, CHCCH_3_C*H*_3_); 1.70 (s, 3H, CHCC*H*_3_CH_3_); ^13^C-NMR (CDCl_3_): 17.7 (*C*H_3_CH_3_C=CH_2_-); 25.7 (CH_3_*C*H_3_C=CH_2_-); 33.5 [(CH_3_)_2_CCH*C*H_2_-]; 115.4 (Ar*C*-3); 115.5 (Ar*C*-5); 120.7 (Ar*C*-5); 123.3 [(CH_3_)_2_C=*C*HCH_2_]; 132.4 [(CH_3_)_2_*C*=CHCH_2_]; 135.1 (Ar*C*-4); 141.3 (Ar*C*-1); 143.4 (Ar*C*-2).

#### 3.2.6. *1,3,5-Trihydroxy-2-(3-methyl-2-buten-1-yl) Benzene* (**13**)

Compound **13** was obtained from phloroglucinol (**6**) as described above. The crude mixture was purified using petroleum ether-ethyl acetate (45:55) as the mobile phase to afford compound **13** as a reddish semi-solid (117.3 mg, 15%); IR (KBr): ν_max_ 3391 (OH), 2974 (C-H alkane), 2926 (C-H alkane), 1616 (C=C aromatic), 1517, 1465, 1375, 1284, 1230, 1144; ^1^H-NMR [(CD_3_)_2_CO]: 7.95 (s, 2H, Ar-1,3-O*H*) 5.93 (s, 2H, Ar*H*-4,6); 5.24 (br. t, 1H, *J* = 7.1 Hz, C=C*H*CH_2_); 3.22 (d, *J* = 7.1 Hz, 2H, C=CHC*H*_2_); 1.71 (s, 3H, CH_3_C*H*_3_C=CH_2_-); 1.60 (s, 3H, C*H*_3_CH_3_C=CH_2_-); ^13^C-NMR [(CD_3_)_2_CO]: 17.5 (*C*H_3_CH_3_C=CH-); 22.2 [(CH_3_)_2_CCH*C*H_2_-]; 25.5 (CH_3_*C*H_3_C=CH-); 95.0 (Ar*C*H-4,6); 106.9 (Ar*C*-2); 125.0 [(CH_3_)_2_C=*C*HCH_2_-]; 129.5 [(CH_3_)_2_*C*=CHCH_2_]; 156.6 (Ar*C*-5); 157.0 ((Ar*C*-1,3).

#### 3.2.7. *1,3-Dihydroxy-4,6-di(3-methyl-2-buten-1-yl) Benzene* (**14**)

Compound **14** was a by-product from the reaction to obtain **9** and was isolated using petroleum ether-ethyl acetate (70:30) as the mobile phase to afford compound **14** as a reddish oil (123.7 mg, 11%); IR (solution): ν_max_ 3419 (OH), 2969, 2914, 2857 (C-H alkanes), 1620 (C=C aromatic), 1507, 1440, 1376, 1300, 1272, 1207, 1162, 1078; ^1^H-NMR (CDCl_3_): 6.78 (s, 1H, Ar*H*-5); 6.33 (s, 1H, Ar*H*-2); 5.29 (br. t, *J* = 6.5 Hz, 2H, 2 × CC*H*CH_2_); 5.10 (s, 2H, Ar-1,3-O*H*) 3.26 (d, *J* = 7.1 Hz, 4H, 2 × C=CHC*H*_2_); 1.77 (s, 6H, 2 × CHCCH_3_C*H*_3_); 1.76 (s, 6H, 2 × CHCC*H*_3_CH_3_); ^13^C-NMR (CDCl_3_): 17.8 (*C*H_3_CH_3_C=CH_2_-); 25.8 (CH_3_*C*H_3_C=CH_2_-); 29.3 [(CH_3_)_2_CCH*C*H_2_-]; 103.7 (Ar*C*-2); 118.7.7 (Ar*C*-4,6); 122.4 [(CH_3_)_2_C=*C*HCH_2_]; 130.9 (Ar*C*-5); 134.4 [(CH_3_)_2_*C*=CHCH_2_]; 153.6 (Ar*C*-1,3).

#### 3.2.8. *1,4-Dihydroxy-2,5-di(3-methyl-2-buten-1-yl) Benzene* (**15**)

Compound **15** was a by-product from the reaction to obtain **10** and was purified using petroleum ether-ethyl acetate (80:20) as the mobile phase to afford compound **15** as a colorless semi-solid, 34 mg, 3%; IR (solution): ν_max_ 3223 (OH), 2978, 2926 (C-H alkanes), 1431, 1373, 1242, 1186; ^1^H-NMR (CDCl_3_): 6.57 (s, 2H, Ar*H*-3,6); 5.28 (br. t, *J* = 7.2 Hz, 2H, 2 × C=C*H*CH_2_); 3.26 (d, *J* = 7.2 Hz, 4H, 2 × C=CHC*H*_2_); 1.76 [s, 12H, 2 × CHC(C*H*_3_)_2_)]; ^13^C-NMR (CDCl_3_): 17.8 (*C*H_3_CH_3_C=CH-); 25.8 (CH_3_*C*H_3_C=CH-); 29.4 [(CH_3_)_2_CCH*C*H_2_-]; 116.9 (Ar*C*-3,6); 121.7 [(CH_3_)_2_C=*C*HCH_2_]; 125.7 (Ar*C*-2,5); 134.6 [(CH_3_)_2_*C*=CHCH_2_]; 147.9 (Ar*C*-1,4).

#### 3.2.9. *1,3-Dihydroxy-5-methyl-4,6-di(3-methyl-2-buten-1-yl) Benzene* (**16**)

Compound **16** was a by-product from the reaction to obtain **11** and was purified using petroleum ether-ethyl acetate (70:30) as the mobile phase to afford compound **16** as a pale yellow semi-solid (174.3 mg, 17%); IR (solution): ν_max_ 3421 (OH), 2969, 2914, 2857 (C-H alkanes), 1601 (C=C aromatic), 1445, 1375, 1324, 1270, 1209, 1158, 1084; ^1^H-NMR (CDCl_3_): 6.23 (s, 1H, Ar*H*-2); 5.12 (br. t, *J* = 6.7 Hz, 2H, 2 × C=C*H*CH_2_); 5.02 (s, 2H, Ar-1,3-O*H*); 3.33 (d, *J* = 6.7 Hz, 4H, 2 × C=CHC*H*_2_); 2.22 (s, 3H, Ar-C*H*_3_); 1.80 (s, 6H, 2 × C*H*_3_CH_3_C=CH_2_-); 1.72 (s, 6H, 2 × CH_3_C*H*_3_C=CH_2_-). ^13^C-NMR (CDCl_3_): 15.8 (Ar-*C*H_3_-5); 17.9 (*C*H_3_CH_3_C=CH-); 25.6 [(CH_3_)_2_CCH*C*H_2_-]; 25.7 (CH_3_*C*H_3_C=CH-); 101.2 (Ar*C*H-2); 118.6 (Ar*C*-4,6); 122.6 [CH_3_)_2_C=*C*HCH_2_]; 133.0 [(CH_3_)_2_*C*=CHCH_2_]; 136.4 (Ar*C*-5) 152.7 (Ar*C*-1,3).

#### 3.2.10. *1,2-Dihydroxy-4,5-di(3-methyl-2-buten-1-yl) Benzene* (**17**)

Compound **17** was a by-product from the reaction to obtain **12** and was purified using petroleum ether-ethyl acetate (70:30) as the mobile phase to afford compound **17** as a reddish semi-solid (198.7 mg, 18%); IR (solution): ν_max_ 3388 (OH), 2970, 2914, 2856 (C-H alkane), 1607 (C=C aromatic), 1514, 1477, 1448, 1375, 1282; ^1^H-NMR (CDCl_3_): 6.66 (s, 2H, Ar*H*-3,6); 5.47 (br. s, 2H, O*H*); 5.20 (br. t, *J* = 6.9 Hz, 2H, 2 × C=C*H*CH_2_); 3.19 (d, *J* = 7.1 Hz, 4H, 2 × C=CHC*H*_2_); 1.72 (s, 6H, 2 × CHCCH_3_C*H*_3_); 1.67 (s, 6H, 2 × CHCC*H*_3_CH_3_); ^13^C-NMR (CDCl_3_): 17.8 (*C*H_3_CH_3_C=CH_2_-); 25.7 (CH_3_*C*H_3_C=CH_2_-); 30.8 [(CH_3_)_2_CCH*C*H_2_-]; 116.1 (Ar*C*-3,6); 123.1 [(CH_3_)_2_C=*C*HCH_2_]; 132.2 [(CH_3_)_2_*C*=CHCH_2_]; 141.3 (Ar*C*-1,2).

#### 3.2.11. *1,3,5-Trihydroxy-2,6-di(3-methyl-2-buten-1-yl) Benzene* (**18**)

Compound **18** was a by-product from the reaction to obtain **13** and was purified using petroleum ether-ethyl acetate (45:55) as the mobile phase to afford compound **18** as a reddish oil (116.7 mg, 11%); IR (solution): ν_max_ 3434 (OH), 2971, 2915, 2857 (C-H alkanes), 1623 (C=C aromatic), 1508, 1449, 1375, 1260, 1226, 1168, 1085; ^1^H-NMR (CDCl_3_): 5.95 (s, 1H, Ar*H*-4); 5.23 (br. t, 2H, *J* = 6.9 Hz, 2 × C=C*H*CH_2_); 3.33 (d, *J* = 7.0 Hz, 4H, 2 × C=CHC*H*_2_); 1.80 (s, 6H, 2 × CH_3_C*H*_3_C=CH_2_-); 1.74 (s, 6H, 2 × C*H*_3_CH_3_C=CH_2_-); ^13^C-NMR (CDCl_3_): 17.8 (*C*H_3_CH_3_C=CH-); 22.3 [(CH_3_)_2_CCH*C*H_2_-]; 25.7 (CH_3_*C*H_3_C=CH-); 96.0 (Ar*C*H-4); 106.2 (Ar*C*-2,6); 122.3 [(CH_3_)_2_C=*C*HCH_2_-]; 134.9 [(CH_3_)_2_*C*=CHCH_2_]; 153.0 (Ar*C*-3,5); 15.0 (Ar*C*-1).

#### 3.2.12. *7-Hydroxy-2,2-dimethyl-chroman* (**19**)

Compound **19**was a by-product from the reaction to obtain **9** and was purified using petroleum ether-ethyl acetate (70:30) as the mobile phase to afford compound **19** as a pale yellow oil (123.8 mg, 15%); IR (solution): ν_max_ 3393 (OH), 2975, 2932, 2852 (C-H alkanes), 1622 (C=C aromatic), 1594, 1508, 1461, 1369, 1296, 1226, 1149, 1119; ^1^H-NMR (CDCl_3_): 6.90 (d, 1H, *J* = 8.2 Hz, Ar*H*-5); 6.38 (dd, 1H, *J_o_* = 8.1 Hz, *J_m_* = 2.4 Hz, Ar*H*-6); 6.34 (d, 1H, *J_m_* = 2.2 Hz, Ar*H*-8); 6.20 (s, 1H, Ar-7-O*H*); 2.70 (t, *J* = 6.6 Hz, 2H, CC*H*_2_CH_2_); 1.79 (t, *J* = 6.8 Hz, 2H, CCH_2_C*H*_2_); 1.34 (s, 6H, CH_2_CH_2_C(C*H*_3_)_2_); ^13^C-NMR (CDCl_3_): 21.6 (*C*H_2_CH_2_C(CH_3_)_2_); 26.7 [CH_2_CH_2_C(*C*H_3_)_2_]; 32.9 [CH_2_*C*H_2_C(CH_3_)_2_]; 74.5 [CH_2_CH_2_*C*(CH_3_)_2_]; 103.7 (Ar*C*H-8); 107.6 (Ar*C*H-6); 113.0 (Ar*C*-4a); 130.0 (Ar*C*H-5) 154.4 (Ar*C*-8a); 154.9 (Ar*C*-7).

#### 3.2.13. *6-Hydroxy-2,2-dimethyl-chroman* (**20**)

Compound **20** was a by-product from the reaction to obtain **10** and was purified using petroleum ether-ethyl acetate (80:20) as the mobile phase to afford compound **20** as a reddish oil, 194.6 mg, 24%; IR (solution): ν_max_ 3387 (OH), 2974, 2931, 2850 (C-H alkanes), 1618 (C=C aromatic), 1492, 1449, 1369, 1452, 1243, 1200; ^1^H-NMR (CDCl_3_): 6.66–6.57 (m, 3H, Ar*H*-5,7,8); 6.13 (s, 1H, Ar-6-O*H*); 2.69 (t, *J* = 6.7 Hz, 2H, CC*H*_2_CH_2_); 1.76 (t, *J* = 6.8 Hz, 2H, CCH_2_C*H*_2_); 1.32 [s, 6H, CH_2_CH_2_C(C*H*_3_)_2_]; ^13^C-NMR (CDCl_3_): 22.5 [*C*H_2_CH_2_C(CH_3_)_2_]; 26.6 [CH_2_CH_2_C(*C*H_3_)_2_]; 32.7 [CH_2_*C*H_2_C(CH_3_)_2_]; 73.9 [CH_2_CH_2_*C*(CH_3_)_2_]; 114.5 (Ar*C*H-5); 115.5 (Ar*C*H-7); 117.6 (Ar*C*H-8), 121.7 (Ar*C*-4a); 147.5 (Ar*C*-8a); 148.6 (Ar*C*-6).

### 3.3. General Procedure for the Acetylation Reactions

To a stirred solution of prenylated phenol (1 equiv.) in methylene chloride (10 mL) was added dimethylaminopyridine (0.1 equiv.) and acetic anhydride (4 equiv.) at room temperature. After 1 h, the solvent was evapored under reduced pressure. Finally, the mixture was subjected to silica gel flash column chromatography (ethyl acetate, petroleum ether) to obtain pure products.

#### 3.3.1. *1,4-Diacetoxy-2-metoxi-5-(3-methyl-2-buten-1-yl) Benzene* (**21**)

Compound **21** was obtained from **8** as described above. The crude mixture was purified using petroleum ether-ethyl acetate (60:40) as the mobile phase to afford compound **21** as a slightly orange solid (67.1 mg, 90%); MS *m/z*: 292 (11%), 250 (22%), 208 (98%), 153 (100%), 69 (9%); mp: 75–77 °C; IR (solution): ν_max_ 1765 (C=O ester), 1621 (C=C aromatic), 1511, 1368, 1206; ^1^H-NMR (CDCl_3_): 6.87 (s, 1H, ArH-6); 6.65 (s, 1H, Ar*H*-3); 5.18 (br. t, *J* = 7.2 Hz, 1H, CC*H*CH_2_); 3.78 (s, 3H, ArC-2-OC*H*_3_); 3.14 (d, *J* = 7.2 Hz, 2H, C=CHC*H*_2_); 2.29 (s, 6H, Ar-1,4-OCOC*H*_3_); 1.73 [s, 3H, CHC(CH_3_) (C*H*_3_)]; 1.67 [s, 3H, CHC(C*H*_3_) (CH_3_)]. ^13^C-NMR (CDCl_3_): 17.7 (*C*H_3_CH_3_C=CH_2_-); 20.6 (OCO*C*H_3_); 20.8 (OCO*C*H_3_); 25.6 (CH_3_*C*H_3_C=CH_2_-); 27.8 (CCH*C*H_2_-); 56.1 (*C*H_3_O-); 107.0 (*C*H-Ar-3); 121.3 (C=*C*HCH_2_); 123.4 (Ar*C*H-6); 125.5 (Ar*C*-4); 133.4 (*C*=CHCH_2_); 137.3 (Ar*C*-1); 146.5 (Ar*C*-5); 149.5 (Ar*C*-2); 168.9 (O*C*OCH_3_); 169.2 (O*C*OCH_3_).

#### 3.3.2. *1,3-Diacetoxy-4-(3-methyl-2-buten-1-yl) Benzene* (**22**)

Compound **22** was obtained from **9** as described above. The crude mixture was purified using petroleum ether-ethyl acetate (70:30) as the mobile phase to afford compound **22** as a colorless semisolid (214.5 mg, 61%); MS *m/z*: 262 (2%), 203 (20%), 178 (20%), 163 (100%), 135 (7%) 107 (9%); IR (solution): ν_max_ 2978 (C-H alkane), 1766 (C=O ester), 1609 (C=C aromatic), 1496, 1422, 1370, 1198 (C-O); ^1^H-NMR (CDCl_3_): 7.22 (d, *J* = 8.4 Hz, 1H, ArH-5); 6.93 (dd, *J* = 8.4 and 2.2 Hz, 1H, ArH-6); 6.86 (d, *J* = 2.2 Hz, 1H, ArH-2); 5.21 (br. t, *J* = 7.2, 1H, CC*H*CH_2_); 3.22 (d, *J* = 7.2 Hz, 2H, CCHC*H*_2_); 2.29 (s, 3H, OCOC*H*_3_); 2.26 (s, 3H, OCOC*H*_3_); 1.74 [s, 3H, CHC(CH_3_) (C*H*_3_)]; 1.69 [s, 3H, CHC(C*H*_3_) (CH_3_)]; ^13^C-NMR (CDCl_3_): 17.7 (*C*H_3_CH_3_C=CH_2_-); 20.7 (OCO*C*H_3_); 20.9 (OCO*C*H_3_); 25.6 (CH_3_*C*H_3_C=CH_2_-); 28.3 [(CH_3_)_2_CCH*C*H_2_-]; 115.8 (Ar*C*-2); 119.0 (Ar*C*-6); 121.3 [(CH_3_)_2_C=*C*HCH_2_]; 130.1 (Ar*C*-5); 130.9 (Ar*C*-4); 133.2 [(CH_3_)_2_*C*=CHCH_2_]; 148.8 (ArC-1); 148.9 (ArC-3); 168.8 (O*C*OCH_3_); 169.0 (O*C*OCH_3_).

#### 3.3.3. *1,4-diacetoxy-2-(3-methyl-2-buten-1-yl) benzene* (**23**)

Compound **23**was obtained from **10** as described above. The crude mixture was purified using petroleum ether-ethyl acetate (80:20) as the mobile phase to afford compound **23** as a colorless oil (115.2 mg, 98%); MS *m/z*: 262 (5%), 219 (17%), 178 (100%), 163 (18%), 123 (36%); IR (solution): ν_max_ 2970 (C-H alkane), 2916 (C-H alkane), 1763 (C=O ester), 1616 (C=C aromatic), 1491, 1438, 1369, 1208 (C-O), 1171 (C-O); ^1^H-NMR (CDCl_3_): 7.02 (d, *J* = 9.5 Hz, 1H, ArH-6); 6.95 (m, 2H, ArH-3,5); 5.21 (br. t, *J* = 7.3 Hz, 1H, C=C*H*CH_2_); 3.22 (d, *J* = 7.2 Hz, 2H, C=CHC*H*_2_); 2.30 (s, 3H, OCOC*H*_3_); 2.28 (s, 3H, OCOC*H*_3_); 1.74 (s, 3H, C*H*_3_CH_3_C=CH-); 1.68 (s, 3H, CH_3_C*H*_3_C=CH-); ^13^C-NMR (100 MHz, CDCl_3_): 17.8 (*C*H_3_CH_3_C=CH-); 20.8 (OCO*C*H_3_); 21.1 (OCO*C*H_3_); 25.7 (CH_3_*C*H_3_C=CH-); 28.6 [(CH_3_)_2_C=CH*C*H_2_-]; 119.9 (Ar*C*H-5); 120.8 [(CH_3_)_2_C=*C*HCH_2_]; 122.7 (Ar*C*H-3) 122.9 (Ar*C*H-6); 133.8 [(CH_3_)_2_*C*=CHCH_2_]; 134.9 (Ar*C*-2); 146.2 (Ar*C*-1); 148.2 (Ar*C*-4); 169.2 (O*C*OCH_3_); 169.4 (O*C*OCH_3_).

#### 3.3.4. *1,3-Diacetoxy-5-methyl-4-(3-methyl-2-buten-1-yl) Benzene* (**24**)

Compound **24** was obtained from **11** as described above. The crude mixture was purified using petroleum ether-ethyl acetate (70:30) as the mobile phase to afford compound **24** as a colorless oil (133.5 mg, 63%); MS *m/z*: 276 (1%), 233 (37%), 192 (35%), 177 (19%), 137 (100%). IR (solution): ν_max_ 2924 (C-H alkane), 1770 (C=O ester), 1618 (C=C aromatic), 1480, 1368, 1198 (C-O); ^1^H-NMR (CDCl_3_): 6.81 (d, 1H, *J* = 1.9 Hz, ArH-6); 6.21 (d, 1H, *J* = 2.0 Hz, ArH-2); 4.98 (br. t, *J* = 6.6 Hz, 1H,C=C*H*CH_2_); 3.22 (d, *J* = 6.6 Hz, 2H, C=CHC*H*_2_); 2.30 (s, 3H, Ar-C*H*_3_); 2.28 (s, 3H, OCOC*H*_3_); 2.26 (s, 3H, OCOC*H*_3_); 1.74 (s, 3H, C*H*_3_CH_3_C=CH_2_-); 1.68 (s, 3H, CH_3_C*H*_3_C=CH_2_-); ^13^C-NMR (CDCl_3_): 17.8 (*C*H_3_CH_3_C=CH-); 19.7 (Ar-*C*H_3_-5); 20.8 (OCO*C*H_3_); 21.0 (OCO*C*H_3_); 25.5 (CH_3_*C*H_3_C=CH-); 25.8 [(CH_3_)_2_CCH*C*H_2_-]; 113.5 (Ar*C*H-2); 120.8 (Ar*C*H-6); 121.3 [(CH_3_)_2_C=*C*HCH_2_]; 129.5 (Ar*C*-4); 131.9 [(CH_3_)_2_*C*=CHCH_2_]; 139.0 (Ar*C*-5); 148.3 (Ar*C*-1); 149.0 (Ar*C*-3); 169.1 (O*C*OCH_3_); 169.2 (O*C*OCH_3_).

#### 3.3.5. *1,2-Diacetoxy-4-(3-methyl-2-buten-1-yl) Benzene* (**25**)

Compound **25** was obtained from **12** as described above. The crude mixture was purified using petroleum ether-ethyl acetate (70:30) as the mobile phase to afford compound **25** as a colorless oil (84 mg, 92%); MS *m/z*: 262 (5.5%), 220 (25%), 178 (100%), 163 (45%), 145 (24%). IR (solution): ν_max_ 2979 (C-H alkane), 2936 (C-H alkane), 1771 (C=O ester), 1607 (C=C aromatic), 1505, 1428, 1372, 1210 (C-O); ^1^H-NMR (CDCl_3_): 7.06 (m, 2H, Ar*H*-3,5); 6.70 (s, 1H, Ar*H*-6); 5.30 (br. t, *J* = 7.4 Hz, 1H, CC*H*CH_2_); 3.34 (d, *J* = 7.8 Hz, 2H, C=CHC*H*_2_); 2.28 (s, 3H, OCOC*H*_3_); 2.27 (s, 3H, OCOC*H*_3_); 1.75 (s, 3H, CHCCH_3_C*H*_3_); 1.69 (s, 3H, CHCC*H*_3_CH_3_); ^13^C-NMR (CDCl_3_): 17.7 (*C*H_3_CH_3_C=CH_2_-); 20.6 (OCO*C*H_3_ × 2); 25.7 (CH_3_*C*H_3_C=CH_2_-); 33.5 [(CH_3_)_2_CCH*C*H_2_-]; 122.1 [(CH_3_)_2_C=*C*HCH_2_]; 123.0 (Ar*C*-3,6); 126.3 (Ar*C*-5); 133.3 [(CH_3_)_2_*C*=CHCH_2_]; 139.9 (Ar*C*-2); 140.6 (Ar*C*-4); 141.7 (Ar*C*-1); 168.3 (O*C*OCH_3_); 168.4 (O*C*OCH_3_).

#### 3.3.6. *1,3,5-Triacetoxy-2-(3-methyl-2-buten-1-yl) Benzene* (**26**)

Compound **26** was obtained from **13** as described above. The crude mixture was purified using petroleum ether-ethyl acetate (45:55) as a mobile phase to afford compound **26** as a colorless oil (207.6 mg, 63%); MS *m/z*: 320 (1%), 277 (38.5%), 235 (50%), 194 (79%), 139 (100%); IR (solution): ν_max_ 2972 (C-H alkane), 2927 (C-H alkane), 1772 (C=O ester), 1620 (C=C Aromatic), 1481, 1430, 1370, 1193 (C-O); ^1^H-NMR (CDCl_3_): 6,82 (s, 2H, Ar*H*-4,6); 5,00 (br. t, 1H, *J* = 6.9 Hz, C=C*H*CH_2_); 3.15 (d, *J* = 6.8 Hz, 2H, C=CHC*H*_2_); 2.27 [s, 6H, ArC-1,3-(OCOC*H*_3_)_2_]; 2.25 (s, 3H, ArC-5-OCOC*H*_3_); 1.71 (s, 3H, CH_3_C*H*_3_C=CH_2_-); 1.66 (s, 3H, C*H*_3_CH_3_C=CH_2_-); ^13^C-NMR (CDCl_3_): 17.8 (*C*H_3_CH_3_C=CH-); 20.8 (‑OCO*C*H_3_); 21.0 (OCO*C*H_3_); 23.7 [(CH_3_)_2_CCH*C*H_2_-]; 25.5 (CH_3_*C*H_3_C=CH-); 113.8 (Ar*C*H-4,6); 120.9 [(CH_3_)_2_C=*C*HCH_2_-]; 123.9 (Ar*C*-2); 132.2 [(CH_3_)_2_*C*=CHCH_2_]; 148.4 (Ar*C*-5); 149.5 (Ar*C*-1,3); 168.6 [(-O*C*OCH_3_)_3_].

#### 3.3.7. *1,3-Diacetoxy-4,6-di(3-methyl-2-buten-1-yl) Benzene* (**27**)

Compound **27** was obtained from **14** as described above. The crude mixture was purified using petroleum ether-ethyl acetate (70:30) as the mobile phase to afford compound **27** as a colorless semisolid (101.5 mg, 43%); MS *m/z*: 330 (1%), 287 (30%), 245 (29%), 191 (100%), 177 (12%) 69 (50); IR (solution): ν_max_ 2973, 2915 (C-H alkanes), 1766 (C=O ester), 1592 (C=C aromatic), 1592, 1496, 1437, 1404, 1369, 1198 (C-O); ^1^H-NMR (CDCl_3_): 7.04 (s, 1H, ArH-5); 6.78 (s, 1H, ArH-2); 5.19 (br. t, *J* = 7.1, 2H, 2 × CC*H*CH_2_); 3.18 (d, *J* = 7.2 Hz, 4H, 2 × CCHC*H*_2_); 2.27 (s, 6H, 2 × OCOC*H*_3_); 1.73 [s, 6H, 2 × CHC(CH_3_) (C*H*_3_)]; 1.69 [s, 6H, 2 × CHC(C*H*_3_) (CH_3_)]; ^13^C-NMR (CDCl_3_): 17.8 (*C*H_3_CH_3_C=CH_2_-); 20.8 (OCO*C*H_3_); 25.6 (CH_3_*C*H_3_C=CH_2_-); 28.5 [(CH_3_)_2_CCH*C*H_2_-]; 116.3 (Ar*C*-2); 121.6 [(CH_3_)_2_C=*C*HCH_2_]; 130.9 (Ar*C*-5); 131.0 (Ar*C*-4,6); 133.1 [(CH_3_)_2_*C*=CHCH_2_]; 146.9 (ArC-1,3); 169.1 (O*C*OCH_3_).

#### 3.3.8. *1,4-Diacetoxy-2,5-di(3-methyl-2-buten-1-yl) Benzene* (**28**)

Compound **28** was obtained from **15** as described above. The crude mixture was purified using petroleum ether-ethyl acetate (80:20) as the mobile phase to afford compound **28** as a colorless semisolid (29.3 mg, 88%); MS *m/z*: 330 (4%), 287 (29%), 246 (100%), 190 (65%), 69 (45%); IR (solution): ν_max_ 2968, 2910, 2857 (C-H alkanes), 1755 (C=O ester), 1498, 1560, 1440, 1366, 1221 (C-O), 1209 (C-O), 1183; ^1^H-NMR (CDCl_3_): 6.85 (s, 2H, Ar*H*-3,6); 5.19 (br. t, *J* = 7.2 Hz, 2H, 2 × C=C*H*CH_2_); 3.17 (d, *J* = 7.1 Hz, 4H, 2 × C=CHC*H*_2_); 2.29 (s, 6H, OCOC*H*_3_) 1.73 (s, 6H, 2 × C*H*_3_CH_3_C=CH-) 1.66 (s, 6H, 2 × CH_3_C*H*_3_C=CH-); ^13^C-NMR (CDCl_3_): 17.8 (*C*H_3_CH_3_C=CH-); 20.9 (OCO*C*H_3_); 25.7 (CH_3_*C*H_3_C=CH-); 28.4 [(CH_3_)_2_CCH*C*H_2_-]; 121.1 [(CH_3_)_2_C=*C*HCH_2_]; 123.2 (Ar*C*-3,6); 132.2 (Ar*C*-2,5); 133.6 [(CH_3_)_2_*C*=CHCH_2_]; 146.4 (Ar*C*-1,4); 169.4 (O*C*OCH_3_).

#### 3.3.9. *1,3-Diacetoxy-5-methyl-4,6-di(3-methyl-2-buten-1-yl) Benzene* (**29**)

Compound **29** was obtained from **16** as described above. The crude mixture was purified using petroleum ether-ethyl acetate (70:30) as the mobile phase to afford compound **29** as a colorless semi-solid (48.5 mg, 62%); MS *m/z*: 344 (1%), 301 (17%), 285 (7%), 259 (42%), 245 (18%), 227 (31%) 205 (100%), 161 (17%), 69 (35%). IR (solution): ν_max_ 2969, 2916 (C-H alkanes), 1768 (C=O ester), 1596 (C=C aromatic), 1447, 1368, 1288, 1222, 1197 (C-O); ^1^H-NMR (CDCl_3_): 6.67 (s, 1H, ArH-2); 4.98 (br. t, *J* = 6.6 Hz, 2H, 2 × C=C*H*CH_2_); 3.23 (d, *J* = 6.5 Hz, 4H, 2 × C=CHC*H*_2_); 2.28 (s, 6H, 2 × OCOC*H*_3_); 2.22 (s, 3H, Ar-C*H*_3_); 1.74 (s, 6H, 2 × C*H*_3_CH_3_C=CH_2_-); 1.64 (s, 6H, 2 × CH_3_C*H*_3_C=CH_2_-); ^13^C-NMR (CDCl_3_): 15.5 (Ar-*C*H_3_-5); 17.9 (CH_3_*C*H_3_C=CH-); 20.9 (*C*H_3_CH_3_C=CH-); 25.6 (OCO*C*H_3_); 26.4 [(CH_3_)_2_CCH*C*H_2_-]; 114.0 (Ar*C*H-2); 121.7 [(CH_3_)_2_C=*C*HCH_2_]; 130.0 (Ar*C*-4,6); 131.7 [(CH_3_)_2_*C*=CHCH_2_]; 137.9 (Ar*C*-5); 149.0 (Ar*C*-1,3); 169.4 (O*C*OCH_3_).

#### 3.3.10. *1,2-Diacetoxy-4,5-di(3-methyl-2-buten-1-yl) Benzene* (**30**)

Compound **30** was obtained from **17** as described above. The crude mixture was purified using petroleum ether-ethyl acetate (80:20) as the mobile phase to afford compound **30** as a colorless oil (99 mg, 92%); MS *m/z*: 330 (0.6%), 288 (9%), 246 (9%), 232 (11%), 190 (100%), 175 (87%), 131 (11%), 91 (15%), 69 (23%); IR (solution): ν_max_ 2971, 2915 (C-H alkanes), 1774 (C=O ester), 1499, 1437, 1370, 1275, 1210 (C-O). ^1^H-NMR (CDCl_3_): 6.93 (s, 2H, Ar*H*-3,6); 5.23 (br. t, *J* = 7.2 Hz, 2H, 2 × CC*H*CH_2_); 3.28 (d, *J* = 7.2 Hz, 4H, 2 × C=CHC*H*_2_); 2.27 (s, 6H, 2 × OCOC*H*_3_); 1.75 (s, 6H, 2 × CHCCH_3_C*H*_3_); 1.68 (s, 6H, 2 × CHCC*H*_3_CH_3_); ^13^C-NMR (CDCl_3_): 17.8 (*C*H_3_CH_3_C=CH_2_-); 20.6 (OCO*C*H_3_ × 2); 25.7 (CH_3_*C*H_3_C=CH_2_-); 30.9 [(CH_3_)_2_CCH*C*H_2_-]; 121.8 [(CH_3_)_2_C=*C*HCH_2_]; 123.1 (Ar*C*H-3,6); 133.3 [(CH_3_)_2_*C*=CHCH_2_]; 138.3 (Ar*C*-4,5); 139.7 (Ar*C*-1,2); 168.5 (O*C*OCH_3_).

#### 3.3.11. *1,3,5-Triacetoxy-2,6-di(3-methyl-2-buten-1-yl) Benzene* (**31**)

Compound **31** was obtained from **18** as described above. The crude mixture was purified using petroleum ether-ethyl acetate (45:55) as the mobile phase to afford compound **31** as a colorless semi-solid (111.0 mg, 60%); MS *m/z*: 388 (0.1%), 345 (26%), 303 (24%), 287 (17%), 271 (39%), 261 (28%), 247 (62%), 205 (100%), 191 (38%), 163 (40%), 151 (40%), 69 (34%); IR (solution): ν_max_ 2972, 2924 (C-H alkanes), 1770 (C=O ester), 1614 (C=C aromatic), 1475, 1423, 1369, 1193 (C-O); ^1^H-NMR (CDCl_3_): 6,82 (s, 1H, Ar*H*-6); 5,00 (br. t, 2H, *J* = 6.7 Hz, 2 × C=C*H*CH_2_); 3.11 (s, 4H, 2 × C=CHC*H*_2_); 2.27 [s, 3H, ArC-3-(OCOC*H*_3_)_2_]; 2.25 (s, 6H, ArC-1,5-OCOC*H*_3_); 1.70 (s, 6H, 2 × CH_3_C*H*_3_C=CH_2_-); 1.67 (s, 6H, C*H*_3_CH_3_C=CH_2_-); ^13^C-NMR (CDCl_3_): 17.8 (*C*H_3_CH_3_C=CH-); 20.5 (-OCO*C*H_3_); 20.8 (-OCO*C*H_3_); 21.0 (OCO*C*H_3_); 24.4 [(CH_3_)_2_CCH*C*H_2_-]; 25.6 (CH_3_*C*H_3_C=CH-); 115.0 (Ar*C*H-6); 121.2 [(CH_3_)_2_C=*C*HCH_2_-]; 124.6 (Ar*C*-2,4); 132.0 [(CH_3_)_2_*C*=CHCH_2_]; 147.3 [(Ar*C*-1,3,5]; 168.7 (-O*C*OCH_3_).

#### 3.3.12. *7-Acetoxy-2,2-dimethyl-chroman* (**32**)

Compound **32** was obtained from **19** as described above. The crude mixture was purified using petroleum ether-ethyl acetate (90:10) as the mobile phase to afford compound **32** as a colorless semisolid (165.2 mg, 41%); MS *m/z*: 220 (9%), 178 (30%), 163 (14%), 123 (100%), 65 (14%); IR (solution): ν_max_ 2976, 2933, 2854 (C-H alkane), 1765 (C=O ester), 1615 (C=C aromatic), 1589, 1499, 1464, 1427, 1307, 1208 (C-O); ^1^H-NMR (CDCl_3_): 7.04 (d, 1H, *J* = 8.2 Hz, Ar*H*-5); 6.55 (dd, 1H, *J_o_* = 8.2 Hz, *J_m_* = 2.1 Hz, Ar*H*-6); 6.51 (d, 1H, *J_m_* = 2.1 Hz, Ar*H*-8); 2.75 (t, *J* = 6.7 Hz, 2H, CC*H*_2_CH_2_); 2.27 (s, 3H, OCOC*H*_3_); 1.79 (t, *J* = 6.7 Hz, 2H, CCH_2_C*H*_2_); 1.33 [s, 6H, CH_2_CH_2_C(C*H*_3_)_2_]; ^13^C-NMR (CDCl_3_): 21.0 (O*C*OCH_3_); 22.0 [*C*H_2_CH_2_C(CH_3_)_2_]; 26.8 [CH_2_CH_2_C(*C*H_3_)_2_]; 32.5 [CH_2_*C*H_2_C(CH_3_)_2_]; 74.4 [CH_2_CH_2_*C*(CH_3_)_2_]; 110.3 (Ar*C*H-8); 112.8 (Ar*C*H-6); 118.5 (Ar*C*-4a); 129.7 (Ar*C*H-5) 149.6 (Ar*C*-7); 154.6 (Ar*C*-8a); 169.5 (O*C*OCH_3_).

#### 3.3.13. *6-Acetoxy-2,2-dimethyl-chroman* (**33**)

Compound **33** was obtained from **20** as described above. The crude mixture was purified using petroleum ether-ethyl acetate (80:20) as the mobile phase to afford compound **33** (210.1 mg, 76%); MS *m/z*: 220 (18%), 178 (100%), 149 (15%), 163 (23%), 123 (62%); IR (solution): ν_max_ 2976, 2933, 2854 (C-H alkanes), 1765 (C=O ester), 1614 (C=C aromatic), 1589, 1499, 1464, 1426, 1370, 1209 (C-O), 1142; ^1^H-NMR (CDCl_3_): 6.78 (m, 3H, ArH-5,7,8); 2.76 (t, *J* = 6.8 Hz, 2H, Ar-C*H*_2_CH_2_); 2.26 (s, 3H, OCOC*H*_3_); 1.78 (t, *J* = 6.8 Hz, 2H, Ar-CH_2_C*H*_2_-C-O); 1.32 [s, 6H, (C*H*_3_)_2_C]; ^13^C-NMR (CDCl_3_): 21.1 (OCO*C*H_3_); 22.6 (Ar-*C*H_2_CH_2_-C-O); 26.8 ((*C*H_3_)_2_C); 32.5 (Ar-CH_2_*C*H_2_-C-O); 74.3 [(CH_3_)_2_*C*]; 117.7 (Ar*C*H-8); 120.2 (Ar*C*H-7); 121.5 (Ar*C*-4a); 121.8 (Ar*C*H-5); 143.2 (Ar*C*-6); 151.7 (Ar*C*-8a); 170.0 (O*C*OCH_3_).

### 3.4. General Procedure to Antioxidant Activity (DPPH Radical Scavenging Activity)

The radical scavenging activity of prenylated compounds and starting materials towards the radical 2,2-diphenyl-1-picrylhydrazyl (DPPH) was measured as described [20] with modifications to adapt the screen for 96-well plates. Stock solutions of compound were prepared in methanol at a concentration of 1 mM (10 mL). Dissolutions (1–200 µM) were prepared from stock solution. Methanol (90 µL), each dissolution (150 µL), and DPPH (60 µL, Sigma-Aldrich) in methanol (0.5 mM), resulting in a final concentration of 0.1 mM DPPH, were added in a 96-well plate. Methanol was used as the blank sample. The mixtures were left for 30 min at room temperature and the absorbances then were measured at 520 nm. Trolox ™ was used as standard antioxidant. The radical scavenging activity was calculated as follows as: % Inhibition = [(blank absorbance − sample absorbance) / blank absorbance] × 100. The mean of three IC_50_ (concentration causing 50% inhibition) values of each compound was determined graphically.

## 4. Conclusions

A concise method has been developed for the synthesis of prenylated phenols via *EAS* condensation of 2 equiv. of prenol with 1 equiv. of phenol using BF_3_ etherate as a catalyst in an organic solvent mixture. This protocol has the advantages of mild conditions and simple procedure. Syntheses of prenylated phenols under different reaction conditions are being studied to produce monoprenylated phenols in high yield and by-products in low yield. Hydroxyl groups in the *ortho* and *para* position in the benzene ring favors the radical scavenging activity. Dialkylated phenols are better antioxidants than monoalkylated phenols. Free hydroxyl groups are essential for antioxidant activity.
